# Reply to: Ultrafast evolution and transient phases of a prototype out-of-equilibrium Mott-Hubbard material

**DOI:** 10.1038/s41467-019-11744-2

**Published:** 2019-09-06

**Authors:** D. Boschetto, M. Weis, J. Zhang, J. Caillaux, N. Nilforoushan, G. Lantz, B. Mansart, E. Papalazarou, N. Moisan, D. Grieger, L. Perfetti, V. L. R. Jacques, D. Le Bolloc’h, C. Laulhé, S. Ravy, J.-P. Rueff, T. E. Glover, M. P. Hertlein, Z. Hussain, S. Song, M. Chollet, M. Fabrizio, M. Marsi, M. Zaghrioui

**Affiliations:** 1 LOA, CNRS, Ecole Polytechnique, ENSTA Paris, Institut Polytechnique de Paris, 181 Chemin de la Hunière et des Joncherettes, 91120 Palaiseau, France; 2 Laboratoire de Physique des Solides, CNRS, Université Paris-Sud, Université Paris-Saclay, 91405 Orsay, France; 3 International School for Advanced Studies SISSA, Via Bonomea 265, 34136 Trieste, Italy; 4 Laboratoire des Solides Irradiés, Ecole Polytechnique-CEA/SSM-CNRS UMR 7642, 91128 Palaiseau, France; 5 Synchrotron SOLEIL, L’Orme des Merisiers, Saint-Aubin, 91192 Gif-sur-Yvette, France; 6 Advanced Light Source, Lawrence Berkeley National Lab, Berkeley, CA 94720 USA; 7 LCLS, SLAC National Accelerator Laboratory, Menlo Park, CA 94025 USA; 8 Laboratoire GREMAN, CNRS-UMR 7347, IUT de Blois, 41029 Blois Cedex, France

**Keywords:** Electronic properties and materials, Phase transitions and critical phenomena

**Replying to** D. Moreno-Mencía et al. *Nature Communications* 10.1038/s41467-019-11743-3 (2019).

In our paper^1^, we reported on the properties of a transient phase in V_2_O_3_ following excitation with ultrafast light pulses. Time-resolved ARPES results provide clear evidence for such non-thermal phase. In addition, time-resolved reflectivity indicates blue-shifted values of the *A*_*1g*_ phonon frequency with respect to equilibrium; and time-resolved X-ray diffraction reveals a transient change in distance between V atoms. Associating the blue-shift of the coherent phonon frequency to the observed non-thermal phase in the material appears a natural choice, further supported by the *ab-initio* calculations presented in the paper, providing a unifying framework for these multiple pieces of experimental information.

In their correspondence, Moreno-Mencia et al. report a different value for the frequency of the A_1g_ mode of V_2_O_3_ and claim they cannot find any appreciable difference between the values extracted from time resolved reflectivity and from Raman spectroscopy.

In our paper, the phonon frequency measured with pump-probe reflectivity (Fig. 3b and c in ref. ^1^) was compared to reference Raman frequencies taken from the literature^2,3^. In order to provide a more precise determination of the frequency blue-shift for the photoexcited system, we performed a detailed Raman spectroscopy study of our specimens, and compared them directly with the time-resolved reflectivity results.

The Raman measurements have been performed on a Renishaw inVia reflex spectrometer, using a 632.8 nm He–Ne laser as excitation source with two possible configurations for the polarization of the scattered light with respect to the incident one, crossed (HV), and parallel (VV). The absolute values of the measured frequencies were carefully calibrated prior to any measurement on the Si T_2g_ phonon mode at 520.5 cm^−1^.

In Fig. 1a we present polarization dependent Raman spectra taken at 295 K from an undoped V_2_O_3_ polished single crystal (c perpendicular to the surface). In the VV geometry the A_1g_ mode becomes clearly visible and is centered at a frequency of 7.4 ± 0.01 THz. In Fig. 1b we present our ultrafast reflectivity data on the same specimen (800 nm for both pump and probe, crossed polarization) and at the same temperature (295 K). The best fit of the coherent oscillations gives 7.7 ± 0.05 THz for the coherent phonon. At 200 K, the A_1g_ phonon frequencies are shifted of ~0.2 THz with respect to 295 K, giving values of 7.55 ± 0.01 THz for Raman and 7.9 ± 0.05 THz for pump-probe (this latter value is in accord with the one published in^1^ at 200K).

**Fig. 1:**
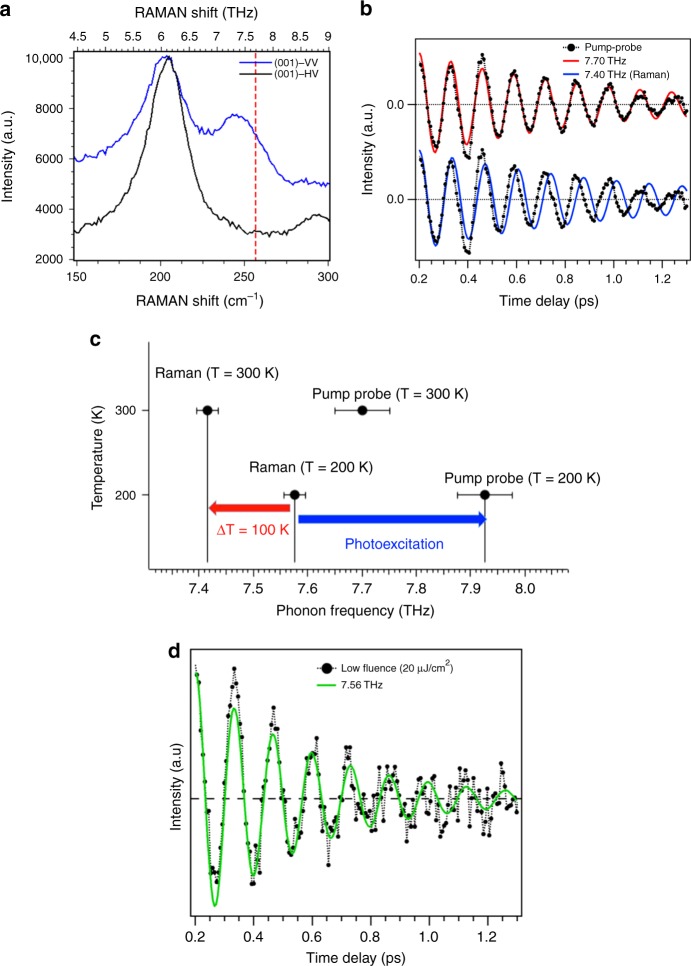
**a** Raman spectroscopy on a V_2_O_3_ single crystal with the c axis perpendicular to the surface at 295 K. The A_1g_ phonon mode is visible at 7.4 THz in the parallel polarization configuration (VV). The vertical dashed line indicates the phonon frequency as derived by fitting the time resolved reflectivity curve in **b**. **b** pump-probe reflectivity at a 8 mJ/cm^2^ fluence on the same specimen in the same experimental conditions, at 295 K. At the top, the background subtracted experimental data are compared with the best sinusoidal fit with a 7.7 THz frequency (in red). At the bottom, the same data are compared to the same sinusoidal function but at 7.4 THz (in blue), corresponding to the Raman frequency. **c** comparison between thermal and pump-probe shifts. At 200 K, the A_1g_ mode frequency is 7.58 THz; by increasing the temperature to 300 K (ΔT = 100 K) the Raman frequency presents a red shift of 0.16 THz; conversely, under ultrafast excitation, it undergoes a blue-shift of 0.35 THz. **d** low fluence measurements performed at 20 μJ/cm^2^ at room temperature; these data were obtained at a repetition rate of 5 MHz. The best fit (green curve) gives a frequency of 7.56 THz

In order to visualize the blue-shift, along with the best fit at 7.7 THz (in red), we present in Fig. 1b also the same coherent oscillating behavior at the Raman frequency (7.4 THz, in blue) superposed to the experimental data: the fact that the pump-probe oscillation frequency is blue-shifted is unambiguous. The sizeable blue-shift between Raman and pump-probe is evident also in Fig. 1a, where the dashed line represents the frequency obtained from the pump-probe data (7.7 THz). We believe this way of comparing the results from the two techniques is more direct and accurate than using FFT procedures, especially when FFT and Raman peaks are very broad.

We repeated the same comparison on several different samples, with different levels of Cr doping and on different surfaces. While we consistently found a sizeable relative blue-shift between pump-probe and Raman results (at least 0.3–0.4 THz), we found that the absolute value of the A_1g_ phonon mode frequency can vary by as much as 0.5–0.6 THz (details in the Supplementary Note 1) depending on material details, such as quality of surface polishing, surface crystallographic orientation, and of course presence of surface defects. The results published in^1^, as well as the ones shown here in Fig. 1, were obtained by polishing the sample as finely as possible (using diamond paste with grains smaller than 1 µm). By using coarser grains to polish the same single crystal, we found smaller frequency values for the phonon mode from pump-probe, down to 7.5 ± 0.05 THz. This pump-probe value is consistent with the one reported by Moreno-Mencia et al.: consequently, it is very likely that the discrepancy between their values of the A_1g_ frequency and our results (published in ref. ^1^ and shown here) is due to different surface polishing. Nevertheless, it should be noticed that our Raman data on the same specimen give a frequency of 7.15 ± 0.01 THz, hence still a clear blue-shift of 0.35 THz.

This dependence on surface sample preparation can be associated to various phenomena, like surface strain or grain size after polishing, and is discussed in the Supplementary information. A complete account of all these aspects is beyond the purposes of this reply, but we would like to point out that the comparison between measurements performed on different samples should be made with caution. In that sense, the shift indicated in Fig. 3b and c of our paper^1^ by using Raman data from the literature should be interpreted as an upper limit for the effect.

Our reply can be summarized in the following points:

First, a sizeable and clearly detectable blue shift between pump-probe and Raman frequencies for the A_1g_ phonon mode can be consistently found in V_2_O_3_ single crystals. The experimental approach detailed above is very clear and direct, and easy to reproduce. It doesn’t need any filtering of the data, and avoids ambiguities in the comparison of very broad FFT peaks and unpolarized Raman spectra (like those presented by Moreno-Mencia et al.).

Second, we emphasize that, out of the dozens of different samples that we measured at fluences of few mJ/cm^2^, never have we found evidence of the red-shift of thermal nature that is routinely found in other materials for the pump-probe frequencies with respect to the Raman ones. This is perfectly consistent with what was previously reported by Misochko et al.^4^, who also found that in V_2_O_3_ the shift always occurs in opposite direction. This is clearly shown in Fig. 1c where the results are summarized for one of the samples: the frequency shift of the pump-probe measurement goes in the opposite direction with respect to the thermal shift, well beyond the experimental error bar.

Third, the question of the fluence dependence of the frequency blue-shift is of course a crucial one. The data presented by Moreno–Mencia in Fig. 2 of their Correspondence cover a fluence window that is irrelevant in this respect. Raman measurements are performed with cw sources with a power of the order of mW. In order to approach this very low excitation regime, we performed measurements on the same sample with fluences of 20 μJ/cm^2^. The results are presented in Fig. 1d: the frequency of the coherent phonon is of 7.56 ± 0.05 THz, clearly reduced with respect to the 7.7 THz observed at 8 mJ/cm^2^. This clearly indicates that it takes even lower fluences to attain the Raman frequency of 7.4 THz. The reduction of the measured frequency shift when reducing the excitation fluence highlights the photoinduced character of this transient state.

Fourth, the results presented here, as well as in our paper^1^, were obtained on carefully prepared samples with a uniform response across their surfaces. This is in stark contrast with the results presented by Moreno–Mencia et al., which appear to come from inhomogeneous surfaces and to depend on not completely controlled sample properties. Our Raman probe had a spot of few μm^2^, and when we explored the lateral dependence of the Raman signal across the various surfaces we studied, we consistently found the same value for the A_1g_ phonon frequency, within the error bar of our measurement (±0.01 THz). We emphasize that only working on laterally uniform samples one can draw sensible conclusions in the comparison of measurements performed with different techniques, like Raman and pump-probe reflectivity.

Finally, both the absolute value of the A_1g_ frequency and the relative value of its blue-shift can vary with various parameters related to material properties and sample preparation, but this doesn’t affect at all the central claims of our paper. The evidence of a non-thermal phase in V_2_O_3_, as well as of an associated transient lattice deformation, comes also from other experimental techniques, and here we fully confirm that it appears natural to associate the A_1g_ hardening to this non-thermal phase.

In conclusion, we performed accurate Raman measurements of the A_1g_ phonon frequency on our samples. By directly comparing our pump-probe measurements with polarization dependent Raman spectroscopy on several specimens in the same experimental conditions, we confirm that a clearly detectable blue-shift can be consistently found between pump-probe and Raman frequencies for the A_1g_ phonon mode in V_2_O_3_. We also demonstrate that the arguments presented by Moreno-Mencia et al. to question the existence of a non-thermal phase in photoexcited V_2_O_3_ are either inconclusive or irrelevant.

This confirms our interpretation that the hardening of the A_1g_ mode is associated to the photoinduced non-thermal phase detected in V_2_O_3_, and all the points in the discussion and in the conclusions of our paper^1^ fully maintain their validity.

## Supplementary information

Supplementary Information

## Data Availability

The data that support the findings of this study are available from the corresponding authors upon reasonable request.
