# Supplementary material for: Reply to: Ultrafast evolution and transient phases of a prototype out-of-equilibrium Mott-Hubbard material
Source: Nat Commun. 2019 Sep 6;10:4035. doi: 10.1038/s41467-019-11744-2 (PMC6731294; doi:10.1038/s41467-019-11744-2)
Supplement: Supplementary file 1 — Supplementary Information [file 41467_2019_11744_MOESM1_ESM.pdf]

SUPPLEMENTARY INFORMATION (Reply by Boschetto et al.)

Supplementary Note 1: Effect of surface polishing on Raman and pump-probe phonon frequencies

In our reply, we discuss how the quality of surface polishing affects the frequency of the A_{1g} mode, both in Raman and pump-probe reflectivity. We present here in Supplementary Figure 1 the data for the coarsely polished V_2O_3 sample discussed in the Reply: the sample was polished with a 4 μm grain size paste, and gives a pump-probe reflectivity frequency comparable to the values presented by Moreno-Mencia et al.

a)

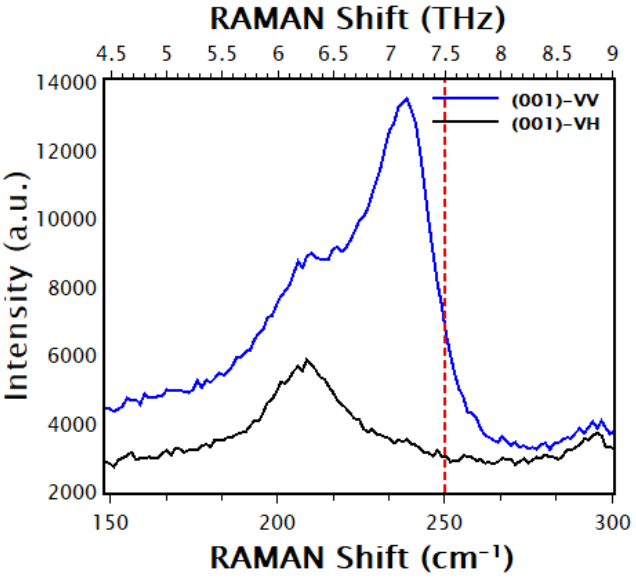

b)

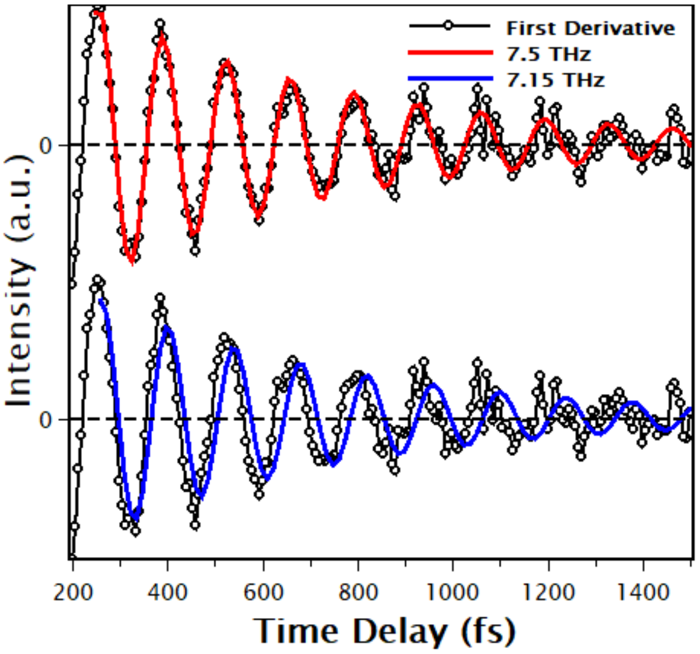

Supplementary Figure 1: *coarsely polished V_2O_3 sample at 295 K: (a) Raman spectroscopy (b) pump-probe reflectivity, compared to the best fit (7.5 THz, in red) and to the same oscillating function but with the Raman frequency (7.15 THz, in blue)*

In Supplementary Figure 2, we also present a summary of the V_2O_3 A_{1g} phonon frequencies measured with Raman spectroscopy for the two surfaces (coarse and fine polishing). The pump-probe data show a relative blue-shift of 0.3-0.5 THz for both specimens at all temperatures.

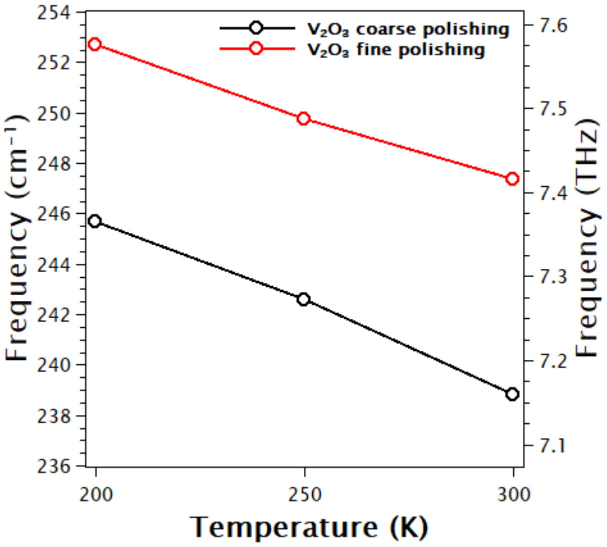

Supplementary Figure 2: *evolution of the Raman A_{1g} frequencies in undoped V_2O_3 with temperature (between 200 K and 300 K) and quality of surface polishing.*

Besides the way polishing is performed during surface preparation, also other effects contribute to variations of the phonon frequencies on V_2O_3. We measured several samples with controlled and uniform properties across their surfaces, and found that the measured phonon frequency can vary on different surface orientations by 0.1-0.2 THz . Even more relevant are the effects of Cr substitution: for instance, the frequency of the A_{1g} phonon in $(V_{1h\ x}Cr_x)_2O_3$ was found to decrease by as much as 0.5-0.6 THz when the doping level goes from x=0 (undoped) to x=0.028.
